# Long non-coding RNA MALAT1 regulates BLCAP mRNA expression through binding to miR-339-5p and promotes poor prognosis in breast cancer

**DOI:** 10.1042/BSR20181284

**Published:** 2019-02-15

**Authors:** Liuhong Zheng, Yuhan Zhang, Yajun Fu, Hangdi Gong, Jianjun Guo, Kangjing Wu, Qiaojun Jia, Xianfeng Ding

**Affiliations:** 1 College of Life Sciences and Medicine, Zhejiang Sci-Tech University, Hangzhou 310018, Zhejiang, China; 2 College of Agriculture and Biotechnology, Zhejiang University, Hangzhou 310012, Zhejiang, China; 3 School Clinics, Zhejiang Sci-Tech University, Hangzhou 310018, Zhejiang, China

**Keywords:** bioinformatics, breast cancer, ceRNA, lncRNA, TCGA

## Abstract

The human genome transcribes a large amount of non-coding RNAs, including long non-coding RNAs (lncRNAs) and microRNAs. LncRNAs and microRNAs have been shown to play a critical regulatory role in tumorigenesis and progression. Competitive endogenous RNAs (ceRNAs) affect other RNAs transcription through competitively binding to common microRNAs (miRNAs). MALAT1 is a typical lncRNA that is markedly up-regulated in breast cancer. However, current understanding of the involvement of MALAT1 in breast cancer development and prognosis remains unclear. In the present study, the expression of MALAT1 in clinical samples of breast cancer tissues was found to be significantly up-regulated that was consistent with the result based on the dataset of the Cancer Genome Atlas (TCGA) at cBioportal. A negative correlation between overall survival and the expression of MALAT1 was statistically significant in the group of diagnosis age below 60 or in the group of infiltrating ductal carcinoma analyzed by TCGA database, which declared that MALAT1 might be a potentially useful prognostic factor. Furthermore, the combination of bioinformatics prediction with experimental verifications indicated that lncRNA MALAT1 can regulate BLCAP mRNA expression through binding to miR-339-5p.

## Introduction

Breast cancer is the most common cancer in women worldwide, with an extremely high mortality rate [1]. It accounts for 25% of all new cancer cases in women, which is the most common cause of death and is second only to lung cancer in female cancer patients in the US [2]. Therefore, it is of paramount importance to understand the pathophysiological mechanisms contributing to breast cancer in order to develop new diagnosis and treatment strategies and improve the overall prognosis of breast cancer patients.

Originally considered as ‘transcription noise’, long non-coding RNAs (lncRNAs) have been recently demonstrated to play important roles in cellular functioning and tumorigenesis through various mechanisms, including post-translational modification, post-translational inhibition and chromatin remodeling, etc. LncRNAs were shown specific aberrant expression in cancer, indicating they may play an important role in biological function in tumor pathogenesis and have a great potential to be used as a prognostic biomarker [3].

MALAT1, metastasis-associated lung adenocarcinoma transcript 1, was also known as nuclear enrichment of the autosomal transcription product 2 (NEAT2). It was highly expressed clinically in many types of cancers such as breast cancer, non-small cell lung cancer and prostate cancer, which indicated that MALAT1 may play an important role in the occurrence and development of tumors [4]. One study found that the level of MALAT1 expression was positively correlated with lymph node metastasis in breast cancer patients and had a significant negative correlation with 5-year disease-free survival (DFS) [5]. However, another study reported that down-regulation of MALAT1 in breast cancer cells induced epithelial–mesenchymal transition (EMT) and promoted the metastasis of breast cancer cells through the PI3K/Akt signaling pathway [6]. Meanwhile, it had a worse prognosis with relatively high expression of MALAT1. Therefore, MALAT1 might have multiple regulatory mechanisms for the metastasis of tumor cells. It is necessary to study more in-depth regulatory mechanisms of MALAT1 in breast cancer, which will help us to understand whether it can be used as a potential diagnostic or prognostic indicator and gene therapy target.

The present study aims to explore the expression level of MALAT1 in breast cancer samples by qRT-PCR and to analyze the clinical significance of MALAT1 using the data from TCGA at cBioportal. To further investigate the molecular regulatory mechanism of MALAT1, bioinformatics predictions were combined with experimental verification. It was found that MALAT1 regulated BLCAP mRNA expression by sponging miR-339-5p. It was the first study demonstrating that MALAT1 might affect BLCAP mRNA expression via regulating miR-339-5p. High expression of MALAT1 might predict poor prognosis in breast cancer.

## Materials and methods

### Preparation of patient samples

A total of seven paired primary breast cancer tissue and corresponding adjacent non-tumor tissue samples were obtained from Zhejiang Cancer Hospital (from 2010 to 2015). No patients received chemotherapy or radiotherapy before the samples were collected. These tissue samples were frozen immediately in liquid nitrogen and stored at −80°C until used. The research has been carried out in accordance with the World Medical Association Declaration of Helsinki. All patients signed informed consent. The present study was approved by the Institutional Research Ethical Committee of Zhejiang Cancer Hospital.

### Collection of publicly available data from TCGA

Level three RNA-seq data and the clinical data were acquired from TCGA breast cancer cohort at cBioportal, consisting of 1105 patients (http://www.cbioportal.org/). The clinical information from the breast cancer cohort of patients logged in TCGA was individually reviewed for overall survival (OS) event times and RNA-Seq data available. In total, 1086 of 1105 patients were chosen for analysis. Clinical and pathological parameters included age, estrogen, progesterone and her2-neu receptor status (ER, PR and Her2), histologic type, tumor size (cm), lymph node status, and distant metastasis status. An institutional review board was not required because all information within TCGA is publicly accessible and de-identified [7].

### Cell culture and treatment

Human breast cancer cell line MCF7 was obtain from Zhejiang University, cultured in DMEM-high glucose medium supplemented with 10% fetal bovine serum (Gibco, Grand Island, NY, U.S.A.) at 37°C in a 5% CO_2_ humidified incubator (Hf 90, Heal force, Hong Kong).

### Construction of interaction network of MALAT1, miRNAs and mRNAs

Using a bioinformatics approach, an lncRNA–miRNA–mRNA regulatory path was constructed. In order to identify if the miRNAs can bind MALAT1, the bioinformatics tool RegRNA2.0 (regrna2.mbc.nctu.edu.tw/detection.html) and BiBiserv2 (bibiserv.cebitec.uni-bielefeld.de) were used. To predict the potential target genes of the screened miRNAs, three databases were utilized: TargetScan (targetscan.org), Microcosm Targets (ebi.ac.uk/Enrightsrv/microcosm/htdocs/targets/v5) and PicTar (pictar.mdcberlin.de/cgi-bin/PicTar vertebrate.cgi). Detailed methods were same as previous study [8]. Finally, the interaction network of MALAT1, miRNA and mRNA was constructed with the cytoscape platform.

### Plasmid construction

The wild-type MALAT1 and BLCAP 3′UTR fragment containing putative binding site of miR-339-5p were amplified by PCR and cloned into the XbaI and NotI sites downstream of Renilla luciferase vector pRL-TK (Promega, Beijing, China); the recombinant plasmids were named pRL-MALAT1-wt and pRL-BLCAP-wt. Similarly, the mutant-type MALAT1 and BLCAP 3′UTR fragment deleted putative binding site of miR-339-5p were amplified by overlap extension PCR and cloned into the XbaI and NotI sites downstream of Renilla luciferase vector pRL-TK; the recombinant plasmids were named pRL-MALAT1-mut and pRL-BLCAP-mut. The primer construction of recombinant plasmids was showed in Supplementary Table S1. Constructs were verified by forward and reverse bidirectional sequencing method.

### Luciferase report assays

Following the manufacturer’s protocol, Lipofectamine™ 2000 (Invitrogen, Carlsbad, CA) was employed for transfection. MCF-7 cells were co-transfected with internal control plasmid pGL3 (Firefly luciferase plasmid) and recombinant plasmid pRL-MALAT1-wt, pRL-MALAT1-mut, pRL-BLCAP-wt or pRL-BLCAP-mut along with mimics for either miR-Ctrl or miR-339-5p. Relative luciferase activity was analyzed at 48 h post-transfection using the dual luciferase reporter assay system (Promega). Renilla luciferase activity was normalized with firefly luciferase activity.

### siRNA and miRNA transfection

Small interfering RNA (siRNA) against MALAT1 (si-MALAT1, 5′-GAGCAAAGGAAGUGGCUUATT-3′), siRNA scrambled control (si-Ctrl, 5′-UUCUCCGAACGUGUCACGUTT-3′), miR-339-5p mimic, miR-339-5p inhibitor and negative control (miR-Ctrl) were chemically synthesized and purified by GenePharma Company (Shanghai, China). The sequences of miR-339-5p mimics, inhibitor and miR-Ctrl are shown in Supplementary Table S4. The transfection was performed according to Lipofectamine 2000 manufacturer’s instruction. Briefly, cells were cultured in a six-well plate to be 50–70% confluent. Prior to transfection, cells were washed with sterile PBS and incubated with serum-free medium for 1 to 2 h. si-MALAT1, si-Ctrl, miR-339-5p mimic, miR-339-5p inhibitor, miR-Ctrl and the transfection reagent were separately mixed with Opti-MEM®I Reduced Serum Medium (Gibco, Grand Island, NY), and the two mixtures were combined and incubated at room temperature for 15 min. The cells were then incubated with the transfection mixture for 4 to 6 h. Then, fresh medium containing 10% FBS was added to the cell culture plates. Finally, cells were harvested and transfection efficiency was examined and detected through the subsequent real-time quantitative polymerase chain reaction (qRT-PCR).

### Quantitative real-time PCR (qRT-PCR)

Total RNA samples were extracted by adopting Trizol reagent (Invitrogen, Carlsbad, CA, U.S.A.), according to the manufacturer’s protocol. RNA purity was measured by Nanodrop-2000. The *A*_260_/*A*_280_ in each RNA sample was above 1.8 and *A*_260_/*A*_230_ above 2.0. The cDNA was synthesized using Reverse Transcriptase M-MLV (TaKaRa Biotechnology Co., Ltd., Dalian, China). qRT-PCR analysis for MALAT1 in tissues, BLCAP in MCF7 cell were performed using the SYBR Green qPCR (Toyobo, Osaka, Japan) according to the manufacturer’s protocol, and the target cDNA was measured using the relative quantification method. GAPDH level was applied as an internal control. For each sample, a comparative threshold cycle (*C*_t_) values were normalized using the formula: Δ*C*_t_ = *C*_t__genes − *C*_t__GAPDH. Relative expression levels were calculated using the formula: ΔΔ*C*_t_ = Δ*C*_t__all_groups − Δ*C*_t__blank control_group. Values used to plot relative gene expression were calculated using the expression, 2^−ΔΔ*C*_t_^. Primers used for cDNA amplification are shown in Supplementary Table S2.

### Statistical analysis

All experiments were repeated at least three times. Data were presented as the means ± standard deviation (SD). One-way analysis of variance was used to pairwise compare different treatment groups of qRT-PCR data.

A patient clinical database from TCGA was established, according to RNA sequencing data, the expression of MALAT1 in breast cancer tissue samples were 395.51–52,899.56. The median was 2846.17. Similarly, the expression of BLCAP in breast cancer tissue samples were 608.76–28,219.97. The median was 1538.63. Using a gene-specific threshold, the expression of each MALAT1 were classified as ‘high expression’ when the expression levels of the MALAT1 were above this threshold and ‘low expression’ if it did not meet the threshold. The gene-specific thresholds were obtained as described by Ramanathan et al. [9].

Clinicopathological parameters, MALAT1 and BLCAP expression levels were separately assigned values, the specific assignment were in Supplementary Table S3. Using the SPSS software version 17.0 for statistics analysis, we analyzed the associations of MALAT1 expression with some clinical characteristics of patients such as ER, PR, Her2, histologic type and tumor size (cm) etc. by Pearson Chi-square analysis or Fisher’s exact test. Significance was accepted for two-sided *P*<0.05. Kaplan–Meier method and Log-Rank test were utilized to describe overall survival (OS) according to MALAT1 expression high or low. Furthermore, for the sub-analyses, we also looked at estrogen, progesterone, her2-neu receptor status (ER, PR and Her2), ages and histologic type etc. Cox proportional hazards regression analyses (univariate and multivariate analysis) were performed to identify whether MALAT1 was the independent factor with a major impact on the overall survival of the patients. OS was measured from diagnosis to last follow-up or death from any cause.

## Results

### MALAT1 was up-regulated in breast cancer tissues

**Figure 1:**
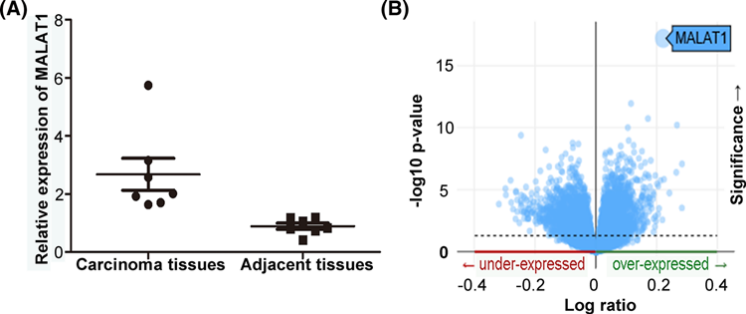
The main titles for Figure 1 or 4 are respectively ‘MALAT1 was upregulated in breast tissues ‘or’ Selection of the microRNA interected with MALAT1 and its targeted gene (**A**) Expression of MALAT1 in breast cancer tissues and corresponding adjacent tissues. MALAT1 expression level in seven paired resected samples was detected by qRT-PCR, normalized with GAPDH. MALAT1 expression was greatly higher in cancer tissues than in corresponding adjacent tissues. (**B**) Expression of MALAT1 in breast cancer tissues from TCGA at cBioportal.

The expression of MALAT1 was measured in seven pairs of breast cancer tissues and adjacent normal tissues. As is shown in Figure 1A, the expression of MALAT1 was significantly higher in breast cancer tissues than that in normal tissues (*P*<0.01). Then, the TCGA database at cBioportal was used to verify the result and came to a similar conclusion (Figure 1B).

### Correlations between MALAT1 expression and clinic-pathological parameters in breast cancer patients

To identify the clinical relevance of MALAT1 expression with breast cancer, publicly available data of breast cancer patients were collected that included RNA-seq data, clinical pathological parameters and survival time from TCGA breast cancer cohort at cBioportal. For the clinic-pathological correlation analysis, 1086 breast cancer patients within TCGA were divided into two groups: high MALAT1 group (*n*=169) and low MALAT1 group (*n*=917) by adopting the gene-specific threshold of MALAT1 expression in breast cancer tissues as a cut-off value. As is shown in Table 1, MALAT1 expression level was significantly correlated with ER status, PR status and diagnosis ages (*P*<0.05), but no significant correlation was identified between MALAT1 expression and HER2 status, tumor size, lymph node status, histological type (*P*>0.05).

**Table 1:** Clinical and pathological characteristics of patients in breast cancer cohort logged in TCGA

Characteristics	Number of cases	MALAT1 expression		χ^2^ value	*P* value^2^
		Low^1^ (*n*=917)	High^1^ (*n*=169)		
Age (years)					
<60	509	415	94		
≥60	576	501	75	6.096	0.014
NA	1	1			
ER status					
Positive	801	652	149		
Negative	235	227	8	32.637	<0.001
NA	50	38	12		
PR status					
Positive	693	564	129		
Negative	340	312	28	19.066	<0.001
NA	53	41	12		
HER2 status					
Positive	164	143	21		
Negative	557	475	82	0.38	0.538
NA	365	299	66		
Tumor size^3^ (cm)					
≤2	279	233	46		
>2	805	682	123	0.23	0.632
NA	2	2			
Lymph node status					
Negative	512	436	76		
Positive	555	466	89	0.29	0.59
NA	19	15	4		
Distant metastasis					
No	900	762	138		
Yes	26	24	2	–-	0.408
NA	160	131	29		
Histologic type					
Infiltrating	202	161	41		
Lobular					
Carcinoma				5.131	0.077
Infiltrating	777	661	116		
Ductal					
Carcinoma	106	94	12		
Other	1				
NA					

NA: missing or indeterminate cases.

^1^The gene-specific thresholds of MALAT1 expression was used as the cutoff. A total of 1086 breast cancer patients within TCGA were divided into two groups: high MALAT1 group (*n*=169) and low MALAT1 group (*n*=917).

^2^For analysis of correlation between the levels of MALAT1 expression and clinical features, Pearson chi-square tests or Fisher exact tests were used. Results were considered statistically significant at two-sided *P*<0.05.

^3^Only the size of invasive tumor is included.

### The impact of MALAT1 expression on prognosis

**Figure 2:**
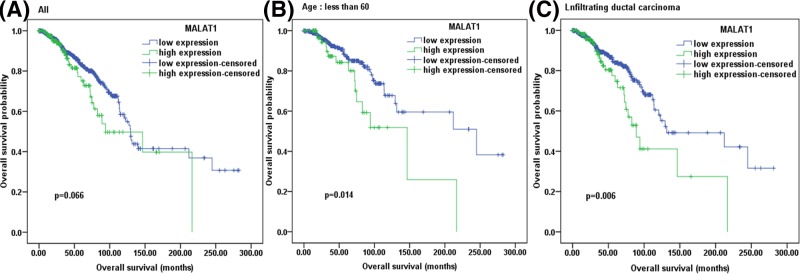
Survival probability for high expression (green) versus low expression (blue) of MALAT1 in breast cancer samples retrieved from TCGA (**A**) Analysis involving all patients: *n*-high = 169; *n*-low = 917. (**B**) Sub-analysis involving the patients of diagnosis age less than 60: *n*-high = 94; *n*-low = 415. (**C**) Sub-analysis involving the patients of infiltrating ductal carcinoma: *n*-high = 116; *n*-low = 661.

The prognostic value of MALAT1 expression for OS was analyzed by Kaplan–Meier analysis and Cox regression analysis (univariate and multivariate analysis). In all patients, there was a correlation between high expression of MALAT1 and a worse survival; however, this correlation was not statistically significant (Figure 2A: *n*-high = 169, *n*-low = 917; *P*=0.066). Therefore, sub-analyses were performed in different sub-groups such as PR-positive, lymph node-negative, no distant metastasis cancers (Supplementary Figure S1). The results were consistent with the conclusion from all patients that were analyzed. Interestingly, a negative correlation between overall survival and MALAT1 expression was statistically significant in the patient group of diagnosis age below 60 (Figure 2B: *n*-high = 94, *n*-low = 415; *P*=0.014) or infiltrating ductal carcinoma (Figure 2C: *n*-high = 116; *n*-low = 661; *P*=0.006), and MALAT1 expression was an independent prognostic factor among them when MALAT1 expression level, ER status, PR status, lymph node status and diagnosis age were included in a multivariate cox regression analysis (Tables 2 and 3).

**Table 2:** Multivariate cox regression analysis of prognostic factors for OS in breast cancer patients according to different histologic types

Variable	Infiltrating lobular carcinoma			Infiltrating ductal carcinoma		
	HR	95% CI	*P* value	HR	95% CI	*P* value
MALAT-1 expression high vs. low	0.67	0.17–2.641	0.567	2.334	1.347–4.046	0.003
ER status positive vs. negative	4.76	0.652–34.734	0.124	0.777	0.37–0.628	0.503
PR status positive vs. negative	1.245	0.23–6.733	0.799	0.686	0.343–1.374	0.288
Lymph node status positive vs. negative	0.202	0.066–0.616	0.005	2.058	1.245–3.403	0.005
Age (years) <60 or ≥60	1.069	1.025–1.114	0.002	2.189	1.396–3.432	0.001

**Table 3:** Multivariate Cox regression analysis of prognostic factors for OS in breast cancer patients according to different age groups

Variable	Age (years) ≥60			Age (years) <60		
	HR	95% CI	P value	HR	95% CI	*P* value
MALAT-1 expression high vs. low	1.059	0.528–2.124	0.872	3.338	1.569–7.104	0.002
ER status positive vs. negative	0.84	0.334–2.108	0.71	0.58	0.247–1.362	0.211
PR status positive vs. negative	0.843	0.364–1.95	0.689	0.436	1.047–0.181	0.063
Lymph node status positivevs. negative	2.282	1.356–3.8442	0.002	1.984	1.056–3.726	0.033

### Selection of the microRNA interacted with MALAT1 and its targeted genes

**Figure 3:**
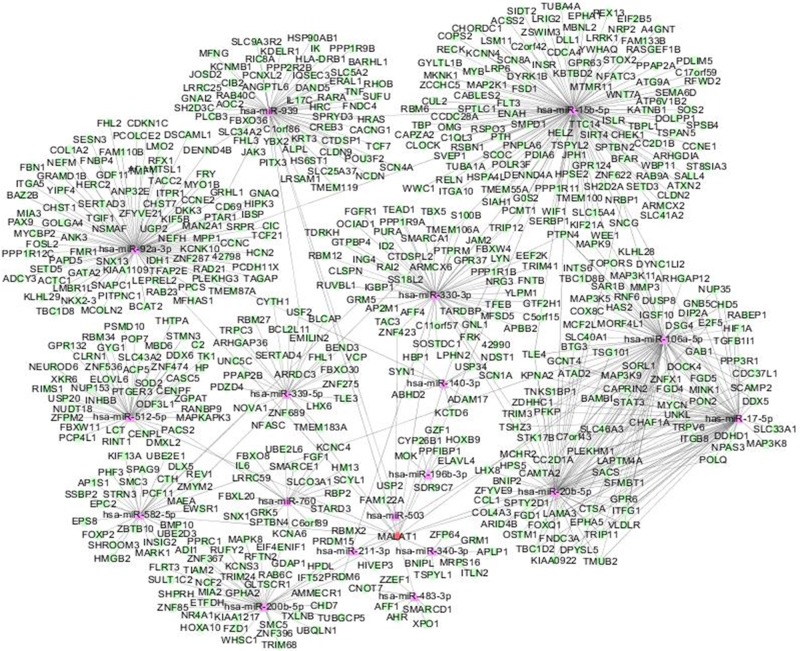
Interaction network of MALAT1–miRNA–mRNA in breast cancer Diamond nodes represent MALAT1, triangle nodes represent miRNAs and circle nodes represent mRNAs. Edges represent the possible associations between MALAT1, miRNAs and mRNA. lncRNA, long non-coding RNA; miRNA, microRNA.

Recently, many lncRNAs were reported to function as sponges to bind specific miRNAs. It was reported that CCAT1 functioned as a molecular sponge for let-7, up-regulated expression of its endogenous targets HMGA2 and c-Myc, and inhibited its function in hepatocellular carcinoma [10]. RoR was also reported to function as a ceRNA to regulate Nanog expression by sponging miR-145 and high expression of RoR predicted poor prognosis in pancreatic cancer [11]. In order to examine whether MALAT1 had a similar mechanism, prediction of miRNAs interacted with MALAT1 and its targeted genes were performed by bioinformatics analysis. We predicted that 18 miRNAs associated with breast cancer might potentially exert regulatory functions on MALAT1 when the minimum folding free energy was set under ≤25 and the system score was set to >160. In order to eliminate false positive rates of the target prediction, only the miRNA–mRNA pairs simultaneously predicted by ≥2 applications were taken forward. A total of 24 genes were predicted to be targets of aberrantly expressed miRNAs. A MALAT1–miRNA–mRNA regulatory network was constructed in the present study. As is presented in the network diagram (Figure 3).

**Figure 4:**
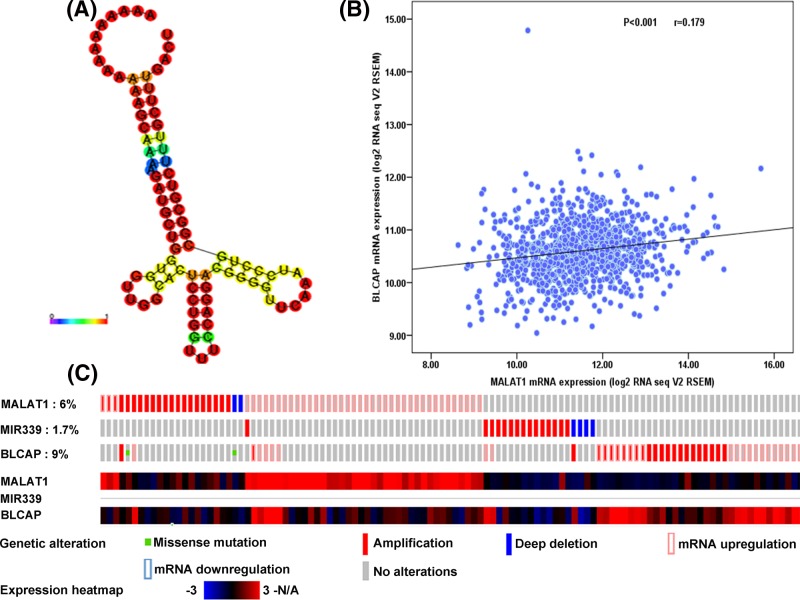
MALAT1 and miR-339-5p RNA fold reliability, expression correlation and differential expression (**A**) RNA fold reliability data of probable long non-coding RNA-microRNA pairs. MALAT1-hsa-miR-339-5P. miR, microRNA. (**B**) MALAT1 transcript and BLCAP mRNA levels were acquired from TCGA breast cancer cohort at cBioportal and mRNA expression (RNA Seq V2 RSEM) of them were subjected to Pearson correlation analysis. (**C**) Identification of differentially expressed MALAT1, miR-339-5p, BLCAP in breast cancer patients from The Cancer Genome Atlas.

Among these 18 microRNAs, miR-339-5p paired with MALAT1 scored highest that suggested the binding capacity of MALAT1 and miR-339-5p might be the strongest. The reliability information of MALAT1 and miR-339-5p pairing structure is depicted in Figure 4A. Moreover, the minimum free energy (MFE) of folding of the structure is −35 kcal/mol that is lowest among 18 miRNAs, suggesting that the possibility of combination between MALAT1 and miR-339-5p is the highest. Besides, the previous study showed that miR-339-5p inhibited breast cancer cell migration and invasion *in vitro* [12] and was also associated with non-small cell lung cancer and lymph node metastases [13]. Furthermore, among target genes of miR-339-5p, as is shown in Figure 4B,C, BLCAP transcript level was significantly correlated with MALAT1 mRNA level and expression alterations of MALAT1, miR-339-5p and BLCAP in breast carcinoma were demonstrated from TCGA at cBioportal. In addition, it has been reported that BLCAP can be regulated by miR-9-3p to promote cell growth and inhibit apoptosis in medullary thyroid carcinoma [14], and there was only one potential complementary site for miR-339-5p in the 3′UTR of BLCAP mRNA. Therefore, we focused on BLCAP as the primary candidate.

### MiR-339-5p is a target of MALAT1

**Figure 5:**
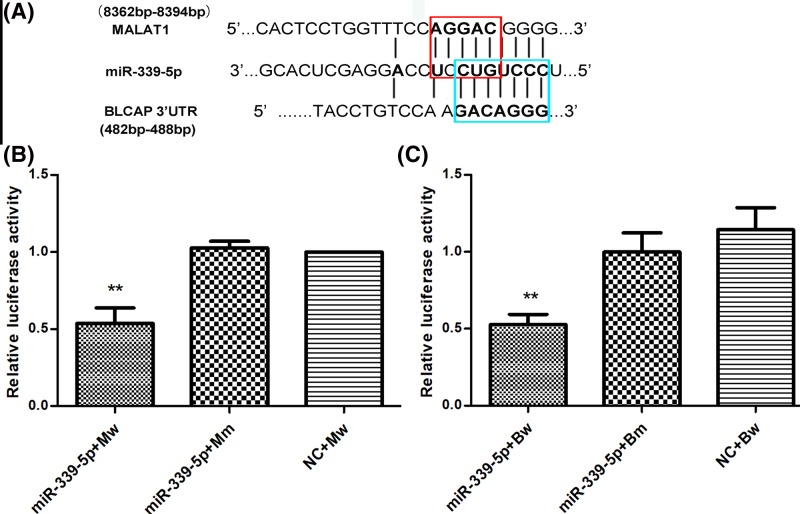
MALAT1 and BLCAP share a common miR-339-5p binding site (**A**) Bioinformatics analysis predicted that MALAT1 and BLCAP share a common miR-339-5p binding site. The red box represents the binding site for miR-339-5p on MALAT1, and the blue box represents the binding site for miR-339-5p on the BLCAP mRNA 3′UTR. (**B** and **C**) Dual luciferase reporter assays results showed that when simultaneously overexpressing miR-339-5p and the MALAT1 8362bp-8394bp sequence in the same cell line, the luciferase activity was significantly lower than that of the control group. Similarly, when simultaneously overexpressing miR-339-5p and the BLCAP mRNA 3′UTR in the same cell line, the Luciferase activity was also significantly lower than that of the control group (**P<0.01).

Through bioinformatics analysis of potential miRNAs of MALAT1 by the online softwares, such as StarBasev2.0, BiBiserve2 and Reg2.0. MALAT1, was found to contain complementary binding sequences to miR-339-5p seed regions (Figure 5A). To explore whether MALAT1 can directly interact with miR-339-5p, luciferase reporter plasmids containing the wild-type or mutated miR-339-5p binding sites in MALAT1 were constructed and co-transfected with miR-control or miR-339-5p mimic into MCF-7 cells. Luciferase reporter assay showed that ectopic expression of miR-339-5p significantly reduced the luciferase activity of MALAT1-WT but not that of MALAT1-MUT (Figure 5B). The data indicated that MALAT1 directly interacted with miR-339-5p.

### MiR-339-5p targeted and regulated BLCAP mRNA expression

We observed the binding site of miR-339-5p on BLCAP 3′UTR through bioinformatics analysis as well as some forecasting softwares like TargetScan, Microcosm Targets and PicTar (Figure 5A). Luciferase assay was employed to validate the regulatory relationship between miR-339-5p and BLCAP. The results suggested that compared with the control cohort, the fluorescence intensity of the co-transfected BLCAP-wt and miR-339-5p mimic group significantly decreased (*P*<0.01), whereas that of the co-transfected BLCAP-mut and miR-339-5p group showed no significant differences (*P*>0.05, Figure 5C), suggesting that there existed a regulatory relationship between BLCAP and miR-339-5p.

**Figure 6:**
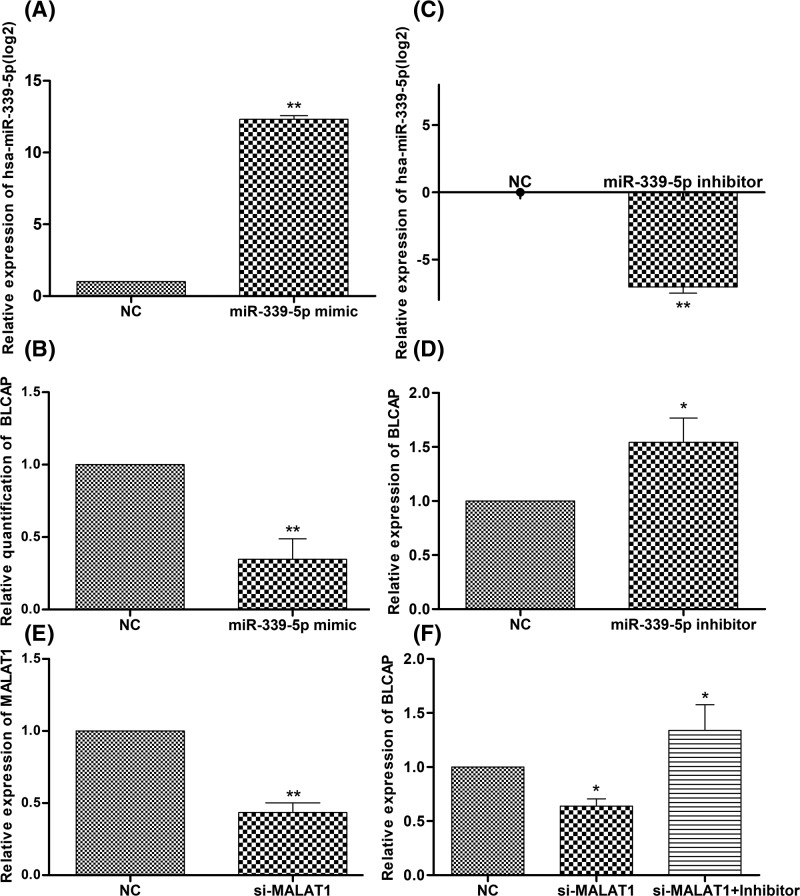
Effects of the MALAT1/miR-339-5P axis on BLCAP mRNA expression in MCF-7 cell (**A**–**D**) qRT-PCR analysis of the expression of miR-339-5p or BLCAP in MCF-7 cells transfected with miRNA mimic or inhibitor. (**E**) qRT-PCR analysis of the expression of MALAT1 in MCF-7 cells transfected with si-MALAT1. (**F**) qRT-PCR analysis of the expressions of BLCAP in MCF-7 cells transfected with si-MALAT1 or si-MALAT1+ miR-339-5p inhibitor (*P<0.05, **P<0.01).

Furthermore, the effects of miR-339-5p on expression of BLCAP mRNA were detected via qRT-PCR. As is shown in Figure 6A–D, compared with the miR-339-5p mimic-Ctrl group, BLCAP mRNA expression was considerably down-regulated after overexpression of miR-339-5p. Conversely, BLCAP expression in the miR-339-5p inhibitor group significantly increased (*P*<0.05). These data indicated that miR-339-5p suppressed the expression of BLCAP mRNA. Meanwhile, the influence of the MALAT1/miR-339-5p axis on BLCAP mRNA expression was examined, BLCAP mRNA expression in the si-MALAT1 group significantly decreased, whereas down-regulated expression of miR-339-5p was noticed to partially reverse the inhibitory effects of MALAT1 on BLCAP mRNA expression (Figure 6E,F). All the above results suggested that MALAT1 regulated the expression of BLCAP mRNA through binding to miR-339-5p in breast cancer.

## Discussion

MALAT1 is a typical lncRNA that plays important regulatory roles in progression of several tumors [15–18]. Chou et al. [19] found that MALAT1 induced migration and invasion of human breast cancer cells by competitively binding miR-1 with cdc42. In addition, some studies indicated that MALAT1 promoted proliferation and invasion via targeting miR-129-5p in triple-negative breast cancer [20]. All these previous studies showed that MALAT1 expression was positively correlated with proliferation, migration and invasion. Meanwhile, patients with higher MALAT1 expression had a worse prognosis in breast cancer. However, some studies have drawn contrary conclusions. For instance, down-regulation of MALAT1 promoted migration and invasion in breast cancer cells and induced EMT by regulating the PI3K–AKT pathway [21]. In summary, the relationship of MALAT1 and breast cancer remains elusive. Therefore, the aim of the present study was to explore the role of MALAT1 in breast cancer oncogenesis and its potential value as a prognostic biomarker.

Here, the expression of MALAT1 in breast cancer samples was examined and the TCGA database at cBioportal was used to verify the results. The results demonstrated that MALAT1 was up-regulated in breast cancer tissues, which was in agreement with previous reports about up-regulation of MALAT1 in non-small cell lung cancer (NSCLC) [22] and hepatocellular carcinoma [23].

The TCGA database brought together high-throughput sequencing or microarray analysis results for a large sample size of each cancer type. More than 1000 breast cancer patient samples collected worldwide were found in TCGA, which were enough for identifying prognostic markers of breast cancer and eliminated the influence of individual variances. Moreover, RNA expression data could also be obtained from high-throughput sequencing using the same platform [24]. In the present study, 1086 of 1105 patients logged in TCGA at cBioportal were chosen for analysis. By analyzing the data from TCGA, MALAT1 expression was found to closely correlate with ER status, PR status and diagnosis age. However, correlation between high MALAT1 expression and poor survival for breast cancer patients was not statistically significant. Many studies have suggested that the prognostic outcome of breast cancer patients was diverse for different age groups, stages and subtypes [25]. Moreover, it has been difficult to find a universal prognostic biomarker. In the clinic, it was important to find a marker with a better prognostic effect in different breast cancer subtypes [3]. Interestingly, for the group of diagnosis age below 60 or infiltrating ductal carcinoma, we found that a correlation between an improved survival with MALAT1 low expression was statistically significant and MALAT1 was an independent prognostic factor among them. These findings implied that MALAT1 might be an important biomarker in predicting the prognosis of breast cancer patients.

After assessing the clinical value of MALAT1 in breast cancer, a MALAT1–miRNA–mRNA regulatory network was constructed using a bioinformatics approach, which should facilitate further experimental studies and may be used to refine biomarker predictions for developing novel therapeutic approaches in breast cancer. Recently, the theory of competitive endogenous RNA reveals a new mode of gene expression regulation. The ceRNA regulatory network revealing in depth the regulatory mechanism of transcriptome level during tumorigenesis is more elaborate and more complex [26]. MALAT1 was reported to act as an endogenous sponge of several RNAs in cancers. For example, MALAT1 functioned as a competing endogenous RNA to regulate MCL-1 expression by sponging miR-363-3p in gallbladder cancer [27]. We assumed that it had similar effects in breast cancer. To investigate the miRNA-related functions of MALAT1 in breast cancer, we chose miR-339-5p as a model miRNA for further studies, with a particular focus on the target gene BLCAP. The present study confirmed that lncRNA MALAT1 modulated BLCAP mRNA expression through functioning as a sponge of miR-339-5p, which reiterated the role of MALAT1 in the tumorigenesis-regulating network.

In terms of the results of present study, some limitations should be carefully considered. First, there was a potential bias because positive results are more likely to be published than negative ones. Although we have tried our best to collect all the available information, some data could still be missing. Second, miRNAs regulate their target mRNAs in different ways, the target mRNAs will be destroyed by miRNA when the miRNAs have perfect or near-perfect complementarity to the 3′UTR of target mRNAs. On the other hand, the miRNAs will only inhibit protein accumulation without affecting mRNA expression levels when they have partial complementary sites in the 3′UTR of target mRNAs [28,29]. Third, the effects of MALAT1 and miR-339-5p on BLCAP protein expression need to be further verified by Western blot. Therefore, further experiments are needed to ascertain these results due to the above limitations.

Despite these limitations, the present study still demonstrated that MALAT1 signature was associated with patient survival, and might potentially be used as a new independent prognostic marker to predict the OS of breast cancer patients for diagnosis age below 60 or infiltrating ductal carcinoma. Besides, our study first elucidated MALAT1–miR-339-5p–BLCAP regulatory network that MALAT1 regulates BLCAP mRNA expression through binding to miR-339-5p. These findings provided great insights into breast cancer initiation and progression, and novel potential therapeutic targets and biomarkers for diagnosis and prognosis for breast cancer. However, more clinical studies on the functional mechanism of the lncRNAs that have not yet been investigated need to be conducted.

## Supporting information

**Supplementary Figure 1:** 

**Supplemental Table S1:** Primers for construction of recombinant plasmids

**Supplemental Table S2:** Primers for qRT-PCR

**Supplemental Table S3:** The specific assignment for Clinicopathological parameters and MALAT1, BLCAP expression levels

## Abbreviations

ceRNA: competitive endogenous RNA
ER: estrogen receptor
Her2: Her2-neu receptor
LncRNA: long non-coding RNA
OS: overall survival
PR: progesterone receptor
TCGA: The Cancer Genome Atlas
