# Supplementary material for: Long non-coding RNA MALAT1 regulates BLCAP mRNA expression through binding to miR-339-5p and promotes poor prognosis in breast cancer
Source: Biosci Rep. 2019 Feb 15;39(2):BSR20181284. doi: 10.1042/BSR20181284 (PMC6379223; doi:10.1042/BSR20181284)
Supplement: Supplementary file 1 [file bsr-39-bsr20181284_Supp1.pdf]

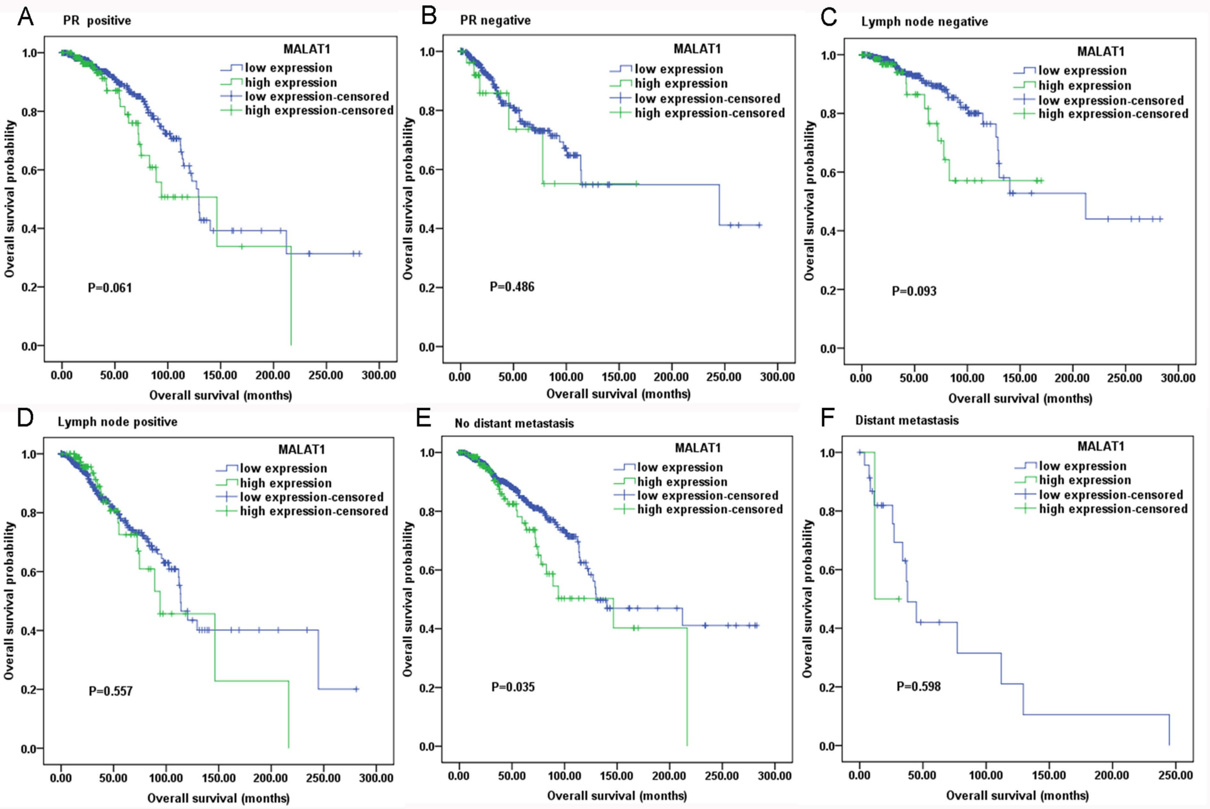

A
PR positive
MALAT1
low expression
high expression
low expression-censored
high expression-censored
P=0.061
Overall survival probability
Overall survival (months)
B
PR negative
MALAT1
low expression
high expression
low expression-censored
high expression-censored
P=0.486
Overall survival probability
Overall survival (months)
C
Lymph node negative
MALAT1
low expression
high expression
low expression-censored
high expression-censored
P=0.093
Overall survival probability
Overall survival (months)
D
Lymph node positive
MALAT1
low expression
high expression
low expression-censored
high expression-censored
P=0.557
Overall survival probability
Overall survival (months)
E
No distant metastasis
MALAT1
low expression
high expression
low expression-censored
high expression-censored
P=0.035
Overall survival probability
Overall survival (months)
F
Distant metastasis
MALAT1
low expression
high expression
low expression-censored
high expression-censored
P=0.598
Overall survival probability
Overall survival (months)

Table .S1. Primers for construction of recombinant plasmids

gene	primer	Sequence (5’-3’)
MALAT1	F	GCTCTAGAATAGGCATTTGAGTGGCTGAGAGG
(wild-type)	R	ATAAGAATGCGGCCGCGCAGTTATATTAGGTTC
MALAT1	R1	GCAGGGATTTGAAGCATCTTTTGCTT
(Mutant)	F2	GCAAAAGATGCTTCAAATCCCTGCGGCG
BLCAP	F	GCTCTAGAAGACGGGAGGGGAGGCATTGA
(wild type)	R	CGGGATCCTTGGGACAGTTGAGAAAGGCA
BLCAP	R1	TGCAAAAATGAGTTGGACAGGTACTCC
(Mutant)	F2	GTACCTGTCCAACTCACTTTTGCACTTATCTCG

F: forward; R: reverse;R1: reverse primer for mutant 1; R2: forward primer for mutant 2

Table .S2. Primers for qRT-PCR

Gene	Sequence	Base number
MiR-339-5p-RT	GTCGTATCCAGTGCAGGGTCCGAGGT ATTCGCACTGGATACGACCGTGAG	50
MiR-339-5p-FP	CGTCCCTGTCCTCCAGGAG	19
MiR-339-5p-RP	AGTGCAGGGTCCGAGGTATT	20
MALAT1-FP	CTCCCCACAAGCAACTTCTC	20
MALAT1-RP	TTCAACCCACCAAAGACCTC	20
BLCAP-FP	GTGAATAATGGCACTGACCG	20
BLCAP-RP	AAGGGAAACCAGAAGAGGGA	20
GAPDH-FP	AACGGATTTGGTCGTATTG	19
GAPDH-RP	GGAAGATGGTGATGGGATT	19

FP: Forward primer RP: Reverse primer

Table .S3. the specific assignment for Clinicopathological parameters and MALAT1, BLCAP expression levels

Influencing factors	Assignment instructions
Age(years)	Less than 60=0 More than 60=1
ER status	Negative=0 positive=1
PR status	Negative=0 positive=1
HER2 status	Negative=0 positive=1
Tumor size (cm) ⁑	≤ 2=0 > 2=1
Lymph node status	Negative=0 Positive=1
Distant metastasis	No=0 Yes=1
Histologic type	Infiltrating Lobular Carcinoma=1<br>Infiltrating Ductal Carcinoma=2<br>Other=3
MALAT1 expression	Low expression=0 high expression=1
BLCAP expression	Low expression=0 high expression=1

ER: estrogen receptor PR: progesterone receptor HER2: human epidermal growth factor receptor 2

⁑Only the size of invasive tumor is included.

Table .S4. The sequences of miR-339-5p mimics, inhibitor and negative control

gene	Sequence (5’-3’)
miR-339-5p mimic	UCCCUGUCCUCCAGGAGCUCACG<br>UGAGCUCCUGGAGGACAGGGAUU
Mimic N.C	UUCUCCGAACGUGUCACGUTT<br>ACGUGACACGUUCGGAGAATT
miR-339-5p inhibitor	CGUGAGCUCCUGGAGGACAGGGA
Inhibitor N.C	CAGUACUUUUGUGUAGUACAA
